# Supplementary figures and images for: Performance of convolutional neural networks for identification of bacteria in 3D microscopy datasets
Source: PLoS Comput Biol. 2018 Dec 3;14(12):e1006628. doi: 10.1371/journal.pcbi.1006628 (PMC6292638; doi:10.1371/journal.pcbi.1006628)

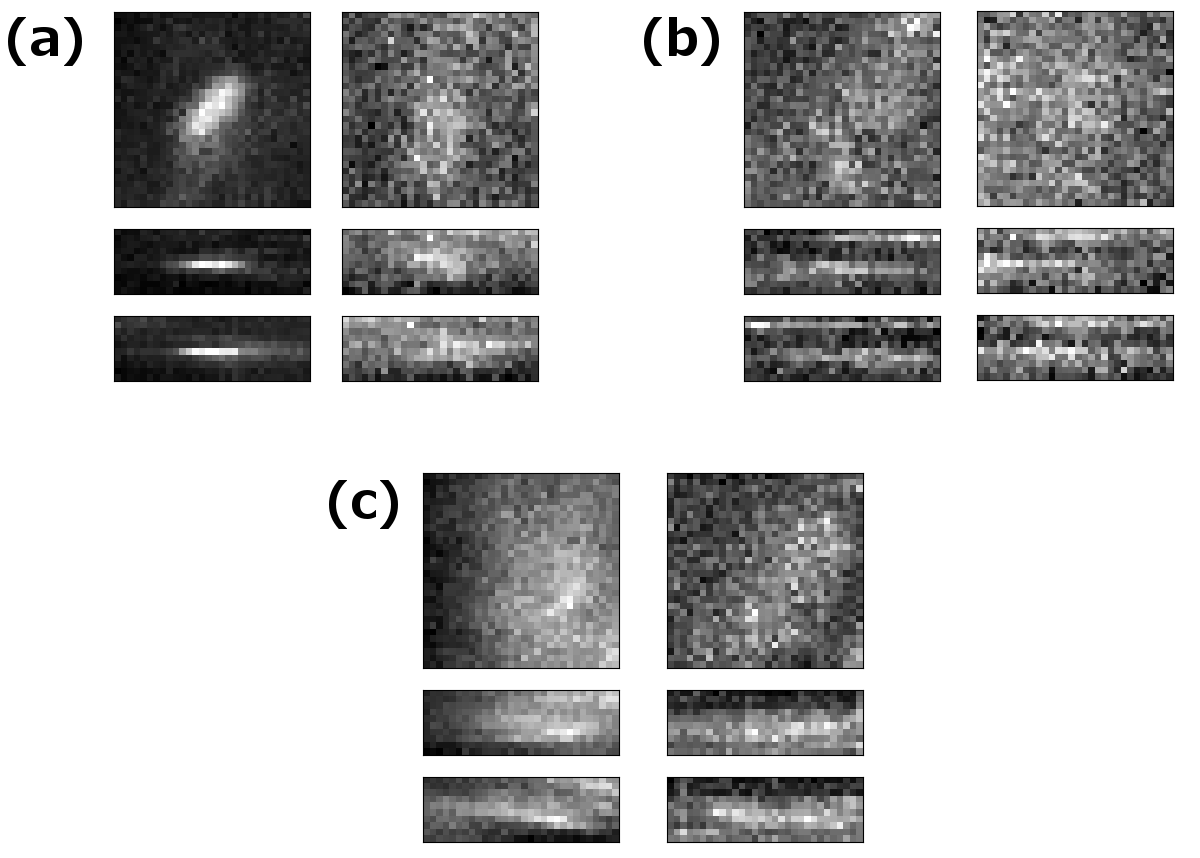

Supplement: S1 Fig — (a) Two example volumes in which all humans classified the object as a bacterium; (b) Two example volumes in which all humans classified the object as noise; (c) Two example volumes in which 50% humans classified the object as a bacterium, and 50% as noise. As in Fig 1, these are xy-, xz-, and yz- projections of the 3D volumes (top to bottom), with the images spanning 4.5 μm in x and y, and 8.0 μm in z. Please note that all 21000 manually classified image volumes, along with labels, are made available for the reader. (TIF) [file pcbi.1006628.s001.tif]
